# Evaluation of polyetheretherketone composites modified by calcium silicate and carbon nanotubes for bone regeneration: mechanical properties, biomineralization and induction of osteoblasts

**DOI:** 10.3389/fbioe.2023.1271140

**Published:** 2023-08-29

**Authors:** Jianfei Cao, Shuhao Yang, Yijun Liao, Yao Wang, Jian He, Chengdong Xiong, Kun Shi, Xulin Hu

**Affiliations:** ^1^ School of Materials and Environmental Engineering, Chengdu Technological University, Chengdu, China; ^2^ Clinical Medical College and Affiliated Hospital of Chengdu University, Chengdu University, Chengdu, China; ^3^ College of Basic Medical and Forensic Medicine, Henan University of Science and Technology, Luoyang, China; ^4^ Chengdu Institute of Organic Chemistry, Chinese Academy of Sciences, Chengdu, China; ^5^ Cancer Center and State Key Laboratory of Biotherapy, Department of Biotherapy, West China Hospital, Sichuan University, Chengdu, China

**Keywords:** polyetheretherketone, carbon nanotubes, calcium silicate, musculoskeletal regeneration, mechanical strength, biocompatibility, biological mineralization

## Abstract

Desired orthopedic implant materials must have a good biological activity and possess appropriate mechanical property that correspond to those of human bone. Although polyetheretherketone (PEEK) has displayed a promising application prospect in musculoskeletal and dentistry reconstruction thanks to its non-biodegradability and good biocompatibility in the body, the poor osseointegration and insufficient mechanical strength have significantly limited its application in the repair of load-bearing bones and surgical operations. In this study, carbon nanotubes (CNT)/calcium silicate (CS)/polyetheretherketone ternary composites were fabricated for the first time. The addition of CS was mainly aimed at improving biological activities and surface hydrophilicity, but it inevitably compromised the mechanical strength of PEEK. CNT can reinforce the composites even when brittle CS was introduced and further upgraded the biocompatibility of PEEK. The CNT/CS/PEEK composites exhibited higher mechanical strengths in tensile and bending tests, 64% and 90% higher than those of brittle CS/PEEK binary composites. Besides, after incorporation of CNT and CS into PEEK, the hydrophilicity, surface roughness and ability to induce apatite-layer deposition were significantly enhanced. More importantly, the adhesion, proliferation, and osteogenic differentiation of mouse embryo osteoblasts were effectively promoted on CNT/CS/PEEK composites. In contrast to PEEK, these composites exhibited a more satisfactory biocompatibility and osteoinductive activity. Overall, these results demonstrate that ternary CNT/CS/PEEK composites have the potential to serve as a feasible substitute to conventional metal alloys in musculoskeletal regeneration and orthopedic implantation.

## 1 Introduction

Over the past few years, the incidence of fortuitous accidents appeared to be an increasing trend, which inevitably leads to bone defects and damages to bone tissues (Salhotra et al., 2020; Dewey and Harley, 2021). In addition, with the aggravation of population aging all over the world, elderly individuals have a higher risk of suffering from bone fractures, imposing heavy burden for the sufferers and society every year (Majidinia et al., 2018). At present, metallic implants are the most widely-used orthopedic materials in surgical operation (Yuan et al., 2020). However, alloy implants will be corroded and may release toxic metal ions *in vivo* (Nielsen, 1987; Su et al., 2019), triggering some postoperative complication linked to the inflammation at the wound site (Prakasam et al., 2017; Pandey et al., 2020). Moreover, elastic modulus of alloy exceeds far more than those of human compact bones and this mismatch also gives rise to bone resorption and osteolysis after surgery (Hussain et al., 2021; Mitra et al., 2021). Therefore, considerable attention was directed towards non-metallic alternatives to minimize adverse post-operation complications and expand application in bone-repairing (Zhao et al., 2021; Hu et al., 2022).

Polyetheretherketone (PEEK) exhibits numerous advantages such as stable chemical resistance, high temperature durability and natural radiolucency (Thiruchitrambalam et al., 2020). Moreover, as an FDA-approved implantable bone substitution material, PEEK has an excellent biocompatibility and appropriate biomechanical properties which are on the verge of natural human bones (Mishra and Chowdhary, 2019; Zhang et al., 2019). Thus, PEEK is logically considered an ideal candidate to replace traditional metal alloy implants in clinical surgery (Haleem and Javaid, 2019; Gao et al., 2021). But essentially, due to its hydrophobic surface, PEEK remains a bioinert and unable to elicit positive interactions with bone tissue (Gu et al., 2021). Long-term clinical observations demonstrated that its implantation might cause a fibrotic encapsulation with connective tissue *in vivo*, isolating the implants from the surrounding osseous tissue (Rousseau et al., 2007; Barkarmo et al., 2013). In the past decades, to increase the applicability of PEEK, the introduction of bioactive calcium phosphate including hydroxyapatite (HA) and β-tricalcium phosphate (β-TCP) into PEEK is a feasible and effective approach (Vaezi et al., 2016; Oladapo et al., 2021). Among a variety of strategies, composite modification usually improves the mechanical modulus and the biocompatibility of PEEK by adjusting parameters of bending and proportion in bioactive fillers (Zheng et al., 2021). However, related studies have shown that the mechanical strengths of binary PEEK composites deteriorate significantly due to the brittleness of fillers and poor interfacial bonding between inorganic particles and PEEK matrix. In some cases, their strength is even lower than that of pristine PEEK (Petrovic et al., 2006; Deng et al., 2015).

As Ca–Si bioceramic, calcium silicate (CaSiO_3_, CS) has already been proven to be biocompatible, biodegradable and bioactive with the osteogenic abilities to stimulate the attachment, proliferation, and differentiation of osteoblast-like cells (Ni and Chang, 2009; Mohammadi et al., 2014). When compared to widely-used HA, CS even demonstrated better bioactivity and bone inductivity in several studies (Jalota et al., 2006). Hu developed mesoporous CS/PEEK composites and discovered that proliferation of MC3T3-E1 cells were improved on composites in comparison with the PEEK, whereas the tensile strength of binary composites decreased (Hu et al., 2016). Ma et al. (2014) prepared CS/PEEK binary composites through a compounding and injection-molding technique and found that the CS/PEEK composite exhibited good bioactivity and biocompatibility, which was confirmed by *in vitro* and *in vivo* tests. As expected, the mechanical strength of the CS/PEEK composite decreased substantially when a high content of fragile CS was added.

Inadequate mechanical strength in load-bearing osseous tissues may cause deformation of orthopedic implants *in vivo*, even if their biological activities meet the requirements of medical physiology. As recognized, carbon nanotubes (CNT) possess extraordinarily high mechanical strength and modulus and have been extensively used to improve the mechanical performance of polymeric composites (Saito et al., 2009; Saito et al., 2014). Furthermore, tissue regeneration can be facilitated by the use of CNT as compatible material. According to the reports of the influence of CNT on osteoblast cells *in vitro*, CNT can promote the proliferation of osteoblasts on their surfaces (Zanello et al., 2006; Li et al., 2008). CNT also showed excellent bone tissue compatibility inducing a minimal inflammatory response and causing little damage to nearby tissue when implanted *in vivo* (Newman et al., 2013). As implant materials applied in clinical operation for arthroplasty or bone fractures already possess exceptional strength, compounding them with CNT to improve the strength properties may yield an effective results. Taken together, the incorporation of CNT enables to overcome the achille’s heel (the decrease in strength) derived from the addition of fragile CS into PEEK and further improves the biocompatibility of PEEK composites.

**SCHEME 1:**
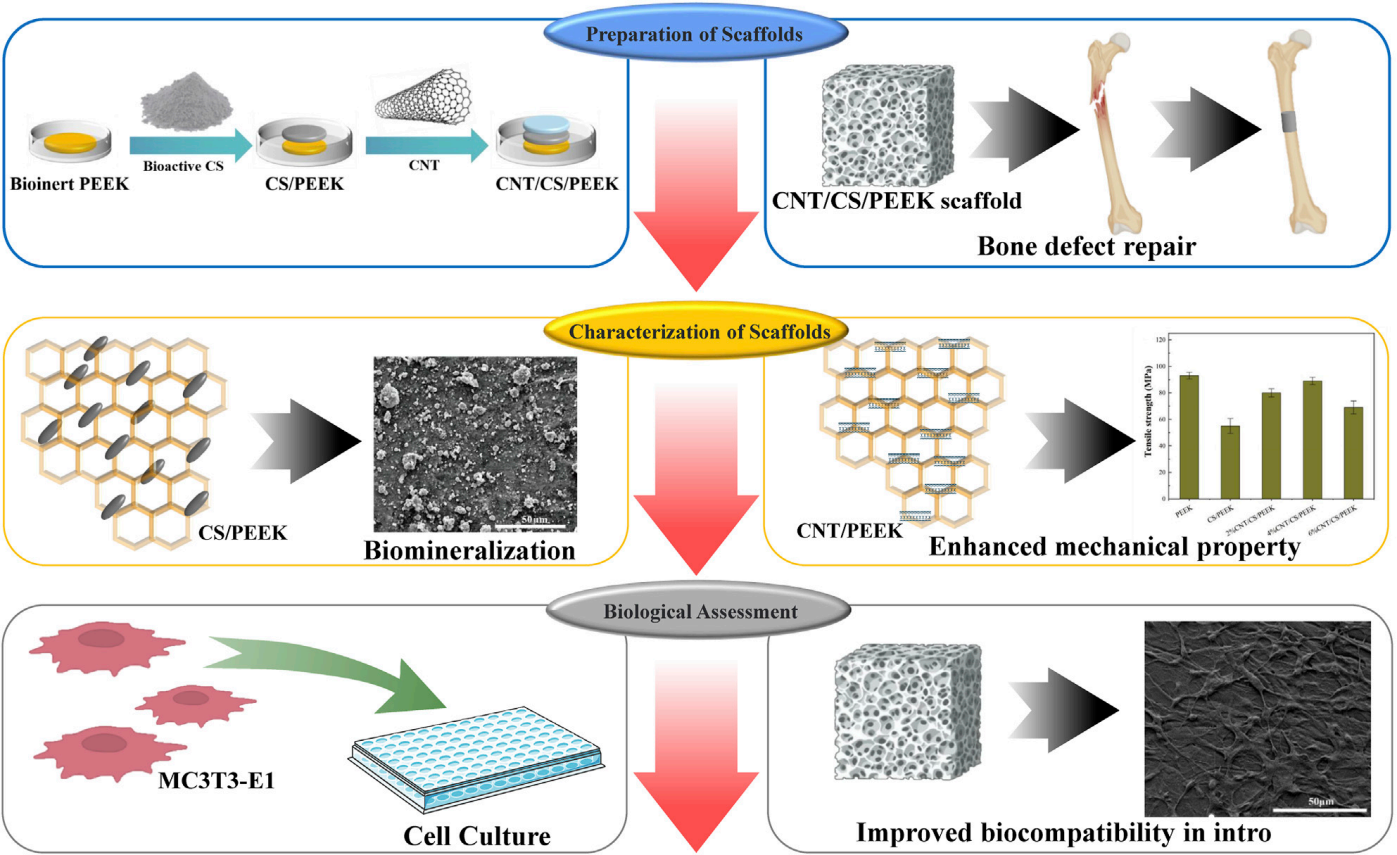
Illustration of the preparation process of PEEK composites modified by CS and CNT for bone regeneration.

In present work, ternary CNT/CS/PEEK composite was fabricated by hybridizing bioactive CS and CNT into PEEK for the first time (Scheme 1). The mechanical properties, morphologies, surface hydrophilicity and roughness of the resulting composites were systematacially studied. In addition, *in vitro* bioactivity and cellular responses of osteoblast to the composite were also evaluated in order to solve the problem of lack of interfacial activity of conventional PEEK implants.

## 2 Materials and methods

### 2.1 Materials

**FIGURE 1:**
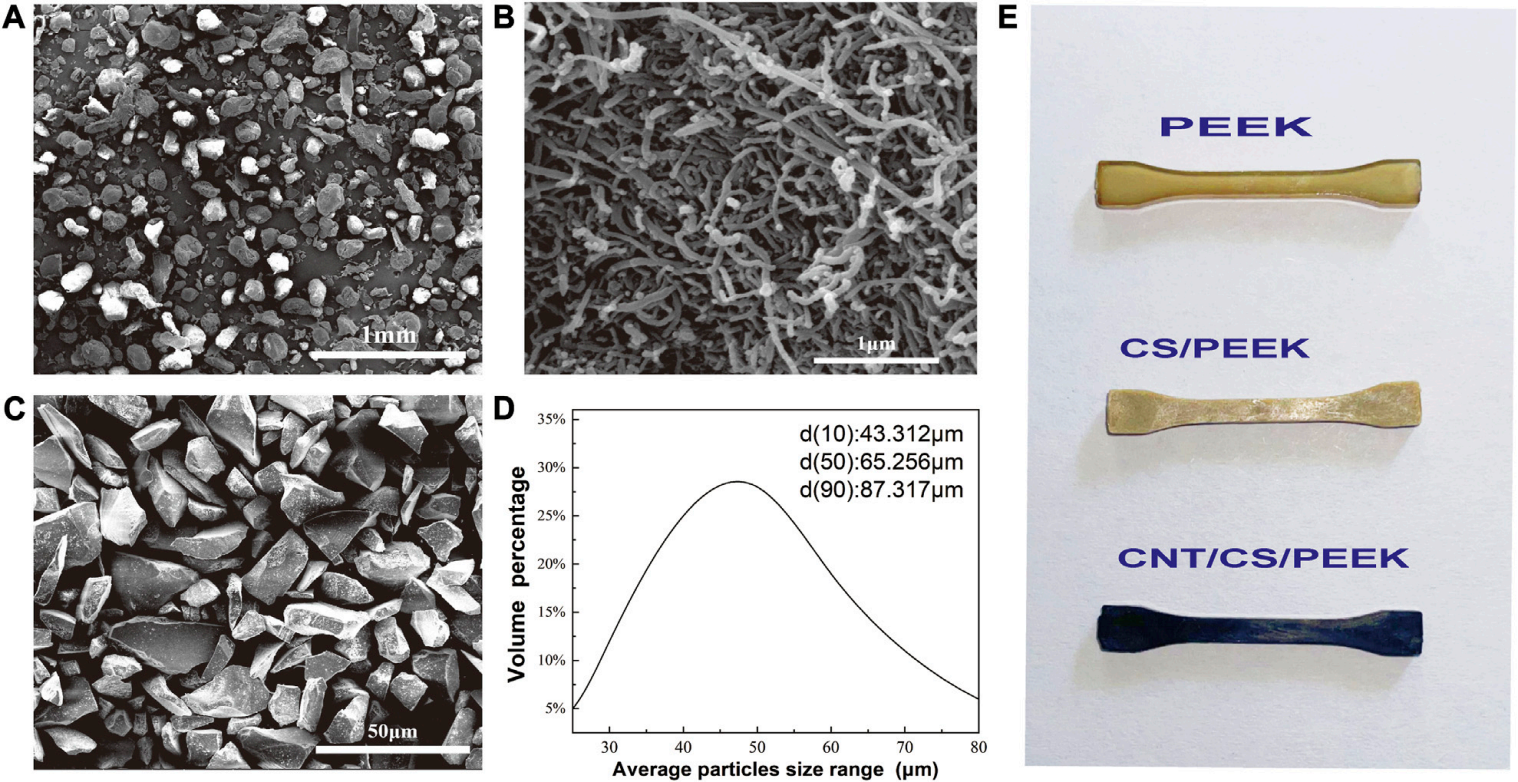
Morphologies of **(A)** PEEK powder; **(B)** CNT; **(C)** Calcium silicate ceramic particles; **(D)** The particle size distribution of CS particles detected by particle size analyzer. **(E)** Standard dumbbell specimens of PEEK composites prepared by injection moulding.

PEEK (Victrex, 450P) was obtained commercially and the average diameter of resin powder was 200 μm (Figure 1A) with a melting point of 350°C. Commercially available CNT (purity ≥96%) were purchased from Chengdu Organic Chemicals Co., Ltd. As shown Figure 1B, the diameter and length of CNT was 30–50 nm and 10–20 μm, respectively. CS particles with irregular shape (Figure 1C) were used as-received from Chinese Technology Co., Ltd. The mean diameter of CS particulates was approximately 65 µm and the granulometric distribution was also shown in Figure 1D, measured by a laser particle analyzer (LS-POP, China).

### 2.2 Preparation of CNT/CS/PEEK composites and specimens

The CNT/CS/PEEK composites with different weight ratios (Table 1) were fabricated by a compounding and injection-molding method as previously reported (Cao et al., 2018). It should be pointed out that in previous literature, the content of CS in PEEK was between 20 wt% and 60 wt%. Although a high content of CS increases bioactivity of composite, it reduces the mechanical strength severely. Hence, in consideration of the balance between mechanical properties and bioactivity, we used the moderate quantity of 30 wt% CS as fillers to modify the PEEK. The similar strategy was also adopted in preparation of other ternary PEEK composites modified by bioactive ceramics (Feng et al., 2016; Abd El-Fattah et al., 2021).

**TABLE 1:** The composing proportion of PEEK, CS/PEEK and CNT/CS/PEEK composites (percentage by weight).

Samples	CNT wt%	CS wt%
PEEK	0	0
CS/PEEK	0	30
2%CNT/CS/PEEK	2	30
4%CNT/CS/PEEK	4	30
6%CNT/CS/PEEK	6	30

CNT aqueous dispersion was prepared in advance according to the method in our previous work using lauryl sodium sulfate (SDS) as dispersant. For each group of composites, quantificational PEEK and CS particles (30 wt%) were dispersed and moistened independently in 150 mL anhydrous ethanol with an ultrasonic treatment for 90 min. After that, two turbid solutions were mixed together and CNT solution that was dispersed beforehand by SDS was guttatim added to the mixture at the dosage of 2 wt%, 4 wt% and 6 wt%. Then an ultrasonic bath and agitation for anther 3 h was carried out. The mixture was filtered and placed in a drying oven at 110°C for 48 h to the constant weight for intensive drying. Finally, to obtain various specimens, the mixture was further molded with an injection-molding machine (BOY-25E, Germany). The melting temperature was 350°C–395°C and the injection pressure was 15–20 MPa. Tensile test was conducted on miniature dumbbell specimen (appearances of dumbbell specimen with different component were shown Figure 1E) and bending test was conducted on rectangular specimen. The circular disks were molded for cell test of MC3T3-E1 cells. All the samples were annealed after rising 20°C–220°C then cooled to room temperature. As a control, PEEK and CS/PEEK composites were also fabricated in accordance with the same preparation technology.

### 2.3 Mechanical properties of composites

The mechanical modulus and strength of the composites were measured by a universal testing machine (SANSCMT4503, China). The tensile test was carried out in ASTMD638 standard and the bending testing was conducted using the test standard of ASTMD790-10. Each group of specimen was tested at room temperature with 5 parallel samples and standard deviation was recorded.

### 2.4 Scanning electron microscopy (SEM) microscopic analysis

A field emission scanning electron microscopy (Phenom pro, Netherlands) was used to observe the microstructure and morphology of the PEEK-based composites. A thin layer of gold coating was sprayed on the on the cross section or surface of the composites to avoid charging effects before observation.

### 2.5 Surface hydrophilicity and surface roughness

A static water contact angle was measured by contact angle goniometer (CA100, HOKUTO, China) at room temperature. 2 μL of distilled water was suspended vertically on the surface of PEEK, CS/PEEK and CNT/CS/PEEK composites with a syringe in different locations for five times. The results were reported in the average values. The roughometer (KEHUI, KH200, China) was used to examine the surface roughness of the composites. Each group was tested using an area of 1,000 μm × 800 µm. Roughness parameters, such as average roughness (Ra), average maximum profile height (Rz) and maximum profile peak height (Rp) were measured using an area of 1,000 μm × 800 µm. For each sample three separate locations were recorded in average values.

### 2.6 Cell culture

MC-3T3E1 (Mouse embryo osteoblast) cells were used for the evaluation of cell compatibility and the proliferation of osteogenic cells to PEEK, CS/PEEK and CNT/CS/PEEK composites. Every sample was sterilized using ultraviolet disinfection prior to cell culture. Osteoblasts were cultured with DMEM medium and digested with 0.25% trypsin solution. Then an appropriate amount of fresh culture medium (10 mL) was added for subculture in a humidified incubator at 37°C and 5% CO_2_. The medium was replaced daily.

### 2.7 Cell adhesion and proliferation

Samples of PEEK, CS/PEEK and CNT/CS/PEEK composites were put into a 96-well cell plate cultured with 1 mL of MC3T3-E1 cells at a density of 1 × 10^4^ cells/well. After being cultured in an incubator at 37°C and 5% CO_2_ for 6 and 12 h, samples were rinsed twice with PBS buffer and stained by AO-EB dye(0.1 g/mL, Sigma, United States) in the dark for 30 min. Fluorescent analysis was used to investigate the cell adhesion by a inverted fluorescence microscope (EVOS M5000, Thermo Fisher, United States).

### 2.8 Cell biocompatibility and metabolic viability

Samples of PEEK and composites were incubated with DMEM medium containing 1% penicillin and 1 mL of MC3T3-E1 cells at a density of 1 × 10^4^ cells/well. Then cells were cultured on the surface of samples in a CO_2_ incubator at 37°C for 1, 4, and 7 days. After each incubation period, specimens were rinsed twice with PBS buffer, 10 μL MTT (5 mg/mL) was added to each well and incubated for another 2 h. After that, cell metabolic viability (the optical density value) was detected by a ELIASA microplate system (SpectraMax, Molecular Devices, China). Five parallel samples were set for each group.

### 2.9 Cellular morphology observed by SEM

The samples were taken out at the corresponding time point of 1, 4, and 7 days and rinsed twice with PBS buffer. Then, each specimen was fixed with 2% glutaraldehyde and dehydrated for 15 min with 30%, 50%, 75%, 85%, 95%, and 100% ethanol (v/v). Finally, samples were dried in drying oven and cell growth on the material surface were observed by SEM.

### 2.10 Bioactivity on biologic biomineralization of composites

The biomineralization assessment of pure PEEK, CS/PEEK and CNT/CS/PEEK composites was evaluated by immersing samples in simulated-body-fluid (SBF) solution, of which the composition and ionic concentration was identical to human tissue fluid. After being soaked in SBF at 37°C for 14 and 28 days, specimens were removed from solution and the formation of bone-like apatite was characterized by an electron microscope equipped with energy dispersive spectroscopy (EDS).

### 2.11 Alkaline phosphatase activity (ALP) assay

The well-grown cells were inoculated with 5 × 10^4^ cells per well and cultured on samples at 37°C and 5%CO_2_ for 7, 10, and 14 days in 96-well plates. Afterwards, each well was treated with a quantitative dose of 4-nitrophenyl phosphate. With the help of alkaline phosphatase, the 4-nitrophenyl phosphate was hydrolyzed into colored 4-nitrophenol. The ALP activity was measured and normalized to optical density value using a microplate system. Five parallel samples were set for each group.

### 2.12 Statistical analysis

A standard deviation was applied to the numerical data in the experiment. One-way analysis of variance (ANOVA) were used for statistical analysis. To be considered statistically significant, the *p*-value of a statistical difference must be less than 0.05.

## 3 Results and discussion

### 3.1 Morphology of composites and distribution of CS particles and CNT in composites

**FIGURE 2:**
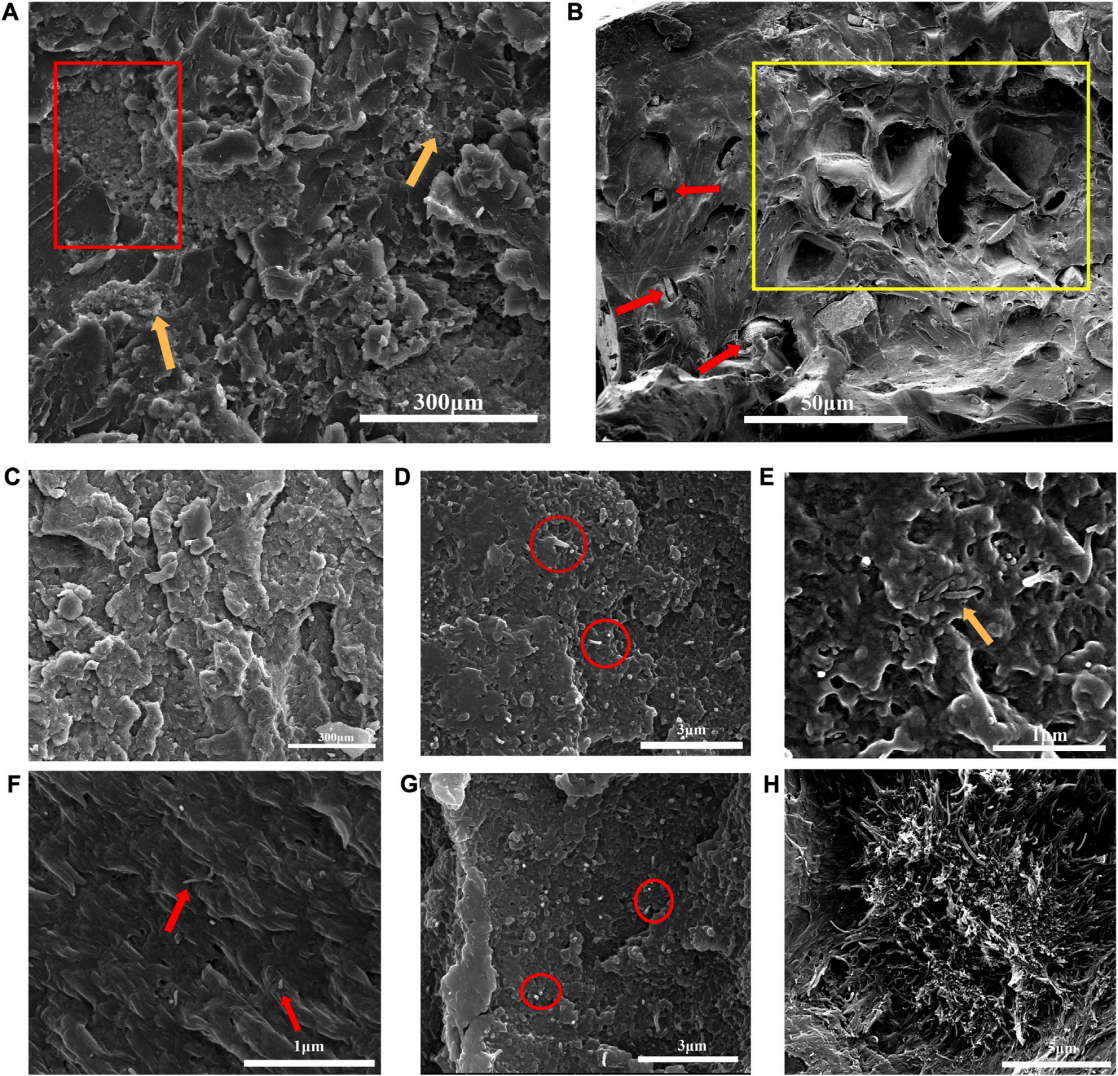
**(A)** Fracture surfaces and distribution of 30 wt% CS particles in CS/PEEK composites observe by SEM; **(B)** Cavities formed by the exfoliation of CS particles. **(C)** Morphology and distribution of 30 wt% CS particles in CNT/CS/PEEK composite; **(D)** Morphology and distribution of CNT in 2%CNT/CS/PEEK composite; **(E)** Entanglement and intersperse of CNT in PEEK polymers; in 4%CNT/CS/PEEK composite **(F)** Efficient interface bonding between CNT and PEEK matrix; **(G)** homogeneous dispersion of CNT in 4%CNT/CS/PEEK composite; **(H)** CNT agglomerated severely in 6%CNT/CS/PEEK composite.

Figures 2A, B revealed the morphology of cross section of binary CS/PEEK composites. A homogeneous distribution of CS particles (30 wt%) was observed in the polymer matrix, which was due to the sufficient mixture of components prior to injection-molding. However, a small amount of CS still agglomerated, which was almost inevitable for inorganic particles in composite materials with high content (pointed by yellow arrows). It was worth noting that the CS particles were stripped by drawing force, leaving some tiny holes in cross-section and making the fracture surfaces rougher (red rectangles). Cavities formed by the exfoliation was also depicted in Figure 2B at high magnification. Although a number of CS particles were embedded in polymers (pointed by red arrows), some cavities left after stripping were evident and conterminous with a diameter ranging from 10 to 50 µm (yellow rectangles). Therefore, the interaction between inorganic CS particles and PEEK was relatively weak and the poor interface adhesion may lead to stress defects when the material was stretched or sheared. Similarly, inadequate interface binding has been reported in other PEEK composites reinforced with different ceramics (Bakar et al., 2003).

The dispersion of CNT and CS fillers in the ternary CNT/CS/PEEK composites was exhibited in Figures 2C–H. The CS particles at a content of 30 wt% were distributed evenly in ternary CNT/CS/PEEK composite as well (Figure 2C). Figures 2D, G showed a homogeneous dispersion of CNT (red circles) without the observable agglomeration with a CNT loading of 2 wt% and 4 wt%, respectively. To achieve a uniform dispersion of carbon tubes is a critical issue in the fabrication of composites to achieve effective reinforcement. Nevertheless it is difficult to accomplish because of a strong tendency of nanotubes to gather and form bundles. As shown in Figure 2F, CNT were not pulled out of the polymer matrix (red arrows) but cut down as no open holes were formed around the nanotubes, which indicated a good interfacial adhesion between CNT and polymer phases. Furthermore, CNT were interspersed and weaved (yellow arrow) through the composites in state of entanglement (Figure 2E). A interconnected and entangled interconnected structure of carbon tubes commonly contributes to reinforcement of resulting composites. However, Figure 2H implied that CNT were not homo-dispersed and began to cluster in larger amounts of 6 wt%. According to relevant literature, the bundles formed by the agglomeration of carbon tubes might seriously reduce the mechanical strength of composites.

**FIGURE 3:**
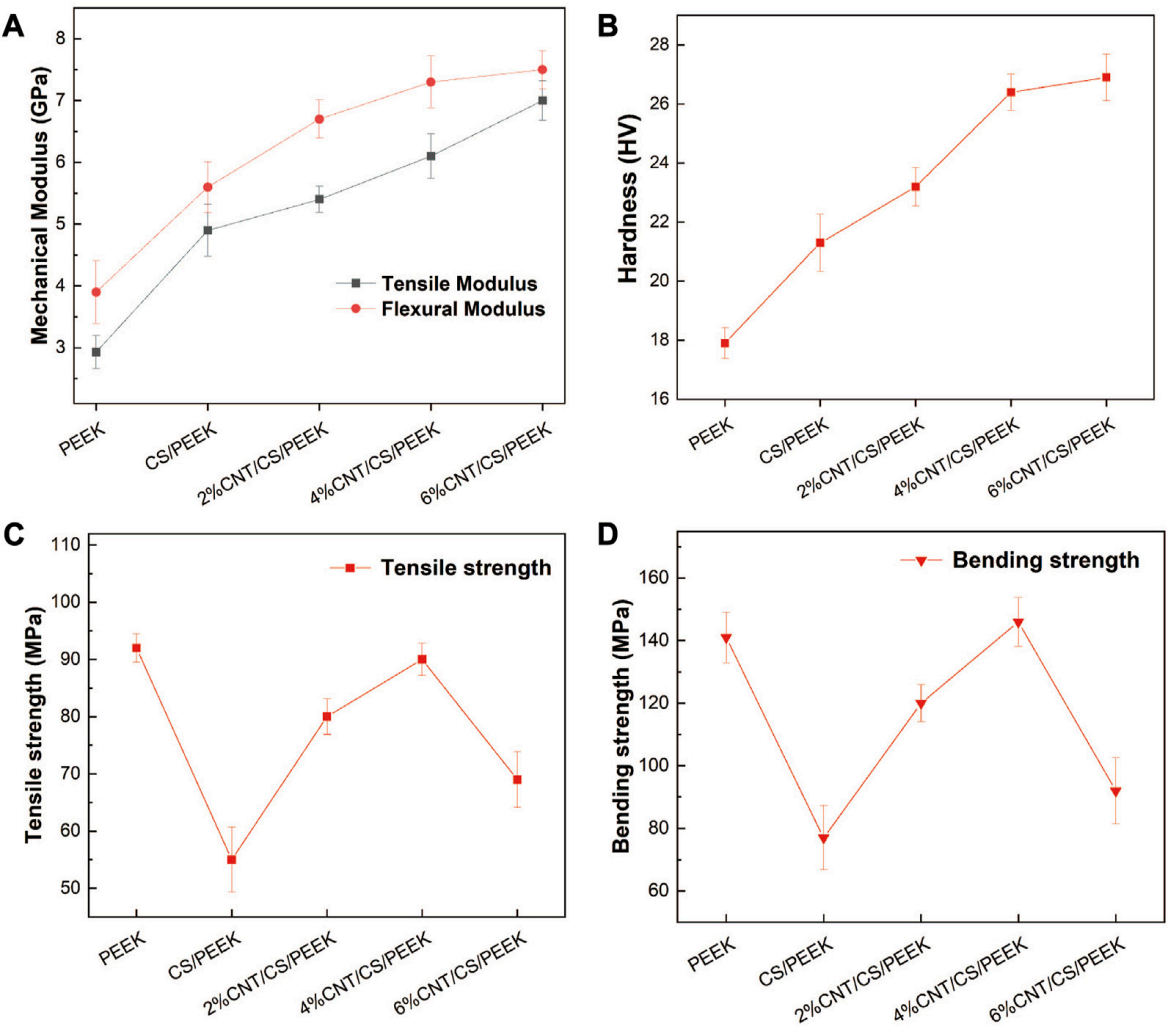
**(A)** Tensile modulus and bending modulus of PEEK and composites; **(B)** Hardness of the PEEK and composites as a function of CS and CNT content. **(C)** Tensile strength and **(D)** Bending strength of PEEK, binary CS/PEEK composite and ternary CNT/CS/PEEK composites.

Figure 3A revealed the variation in mechanical modulus (tensile modulus and bending modulus) of the composites with CS and CNT content. The addition of 30 wt% CS particles increased the tensile modulus and bending modulus to 4.92 GPa and 5.63 GPa, being about 67% and 44% higher than those of PEEK. In addition, tensile modulus and bending modulus of composites generally increased with increasing CNT content. The addition of CNT of 6 wt% improved the tensile modulus and bending modulus of composites with an maximum of 7.13 GPa and 7.50 GPa, which were increased by 143% and 92% when compared with pristine PEEK. The superficial micro-hardness of materials plays a crucial role on the osseointegration and long-term implantation of dental implants. The micro-hardness of the composites was shown in Figure 3B. Due to the higher stiffness and surface micro-hardness of inorganic fillers, the micro-hardness generally enhanced with ceramics filler content in polymer composites. An addition of 30 wt% CS fillers increased the micro-hardness of PEEK to 36 HV, which was 19% higher than that of pure PEEK. Moreover, a further addition of CNT resulted in a statistically significant increase in micro-hardness as well because a given volume fraction CNT exhibited stronger resistance against indentation within the matrix.

The tensile strength and bending strength of composites were presented in Figures 3C, D. Tensile strength and bending strength of pure PEEK were 93 MPa and 141 MPa, respectively. When 30 wt% CS was added to PEEK, the tensile strength and bending strength of binary CS/PEEK composite decreased dramatically with the reduction rates of 41% in tensile strength and 45% in bending strength. Such a result has been widely reported in related papers due to the brittleness and fragility of CS itself. A weak interaction bonding (shown in Figure 2B) between inorganic CS fillers and polymers resulted in a disruption of stress conduction. Notably CNT (with a amount of 2 wt% to 4 wt%) improved both the tensile and bending strength of composites, offering an ample compensation for the mechanical loss caused by the incorporation of friable CS. The tensile and bending strengths of 4%CNT/CS/PEEK came up to 89 MPa and 143 MPa, increased by 61% and 85% when compared to CS/PEEK composite, of which the strengths were within range of human compact bone (Roeder et al., 2008; Hannink and Arts, 2011). The bending strength of 4%CNT/CS/PEEK was even greater than PEEK, indicating a positive reinforcement of carbon nanotubes on strength. However, it was a pity that 6wt% CNT resulted in a sharp decrease in mechanical strength which was explained by agglomeration of CNT, already mentioned in Figure 2H.

**FIGURE 4:**
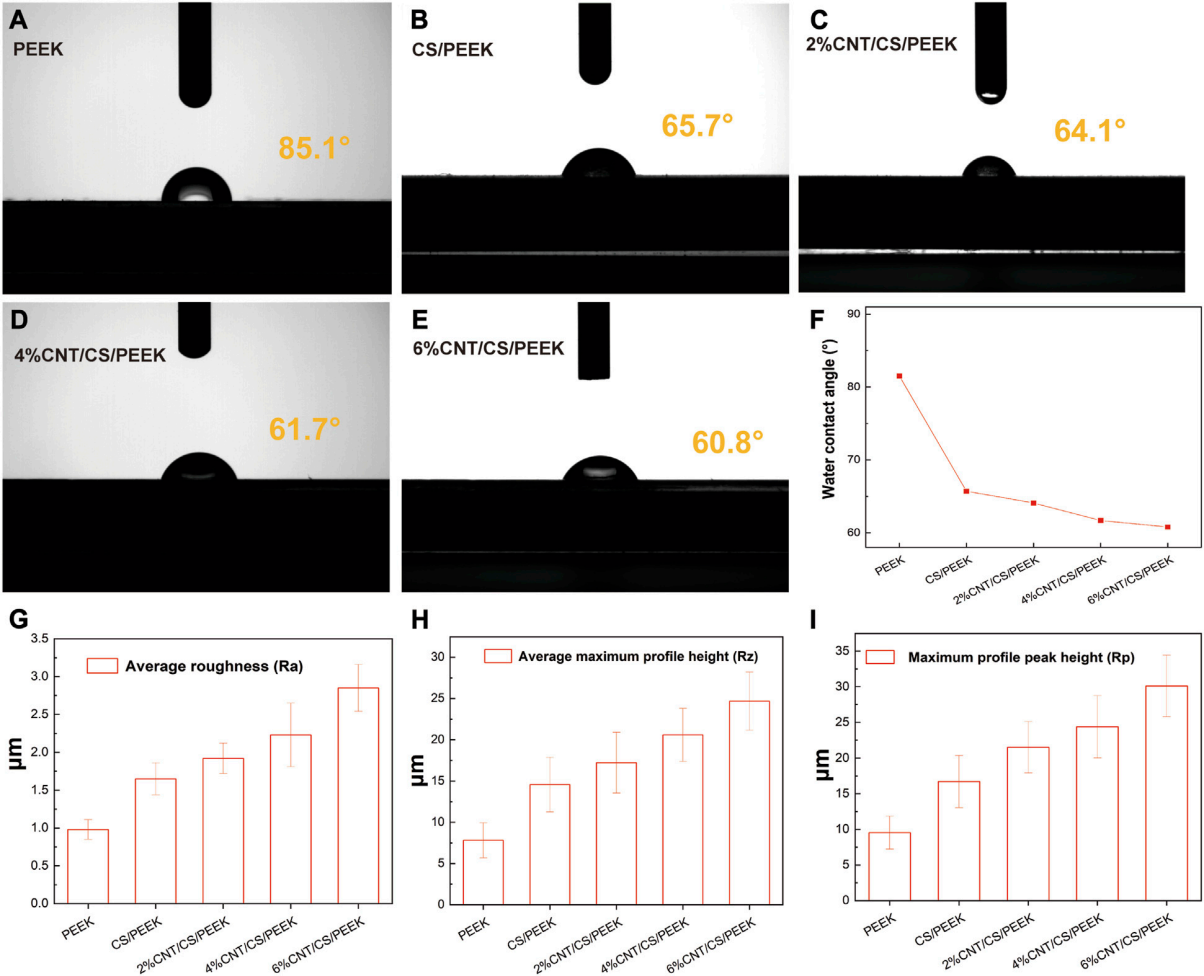
Water contact angles of **(A)** pristine PEEK; **(B)** CS/PEEK; **(C)** 2%CNT/CS/PEEK; **(D)** 4%CNT/CS/PEEK; **(E)** 6%CNT/CS/PEEK; **(F)** Variation trend of water contact angle of PEEK and composites; Measurement of roughness parameters of PEEK and PEEK composites: **(G)** average roughness (Ra), **(H)** average maximum profile height (Rz), **(I)** average profile peak height (Rp).

The hydrophilicity of surface is an important property for estimating the biocompatibility of materials and it is usually characterized by measuring the surface contact angle of samples. Figures 4A–E showed the photographs of contact angles for each sample and trend of water contact angle was also shown clearly in Figure 4F. The contact angles of PEEK was 85.1°, having a slightly hydrophilic surface. Several studies have confirmed that compared to hydrophobic surface, adhesion and proliferation of osteoblasts are more favorable on hydrophilic surface (Mei et al., 2019), which is critical for cell anchorage. While the water contact angles of CS/PEEK decrease to 65.7°, which indicated that the addition of 30 wt% CS significantly improved the hydrophilicity of PEEK. After being incorporated into CNT, water contact angles of 2%CNT/CS/PEEK, 4%CNT/CS/PEEK and 6%CNT/CS/PEEK were declined to 64.1°, 61.7°, and 60.8°, respectively. The surface hydrophilicity of the composites increased slightly with the increasing CNT content.

Surface roughness of PEEK, CS/PEEK and CNT/CS/PEEK composites were summarized in Figures 4G–I. Surface roughness has a significant influence on cells proliferation and function because a rougher surface promoted the cell attachment and differentiation of osteoblasts cultured on surface. The average roughness (Ra) of CS/PEEK composite (1.65 ± 0.21 μm) was 68% higher than PEEK (0.98 ± 0.13 μm) after embedded with coarse CS fillers. Additionally, CNT/CS/PEEK composites with CNT contents (2 wt%-6 wt%) had an increase in surface roughness. For the 6%CNT/CS/PEEK specimen, Ra values markedly increased to (2.85 ± 0.31 μm), almost three times as value of PEEK. Other roughness parameters of average maximum profile height (Rz) and average profile peak height (Rp) were greatly improved after adding CS and carbon nanotubes, following the same pattern of Ra. It is thought that the higher surface roughness was caused by the CNT and CS filler exposed on the PEEK matrix surfaces, which alters the surface morphology and microstructure of surface.

**FIGURE 5:**
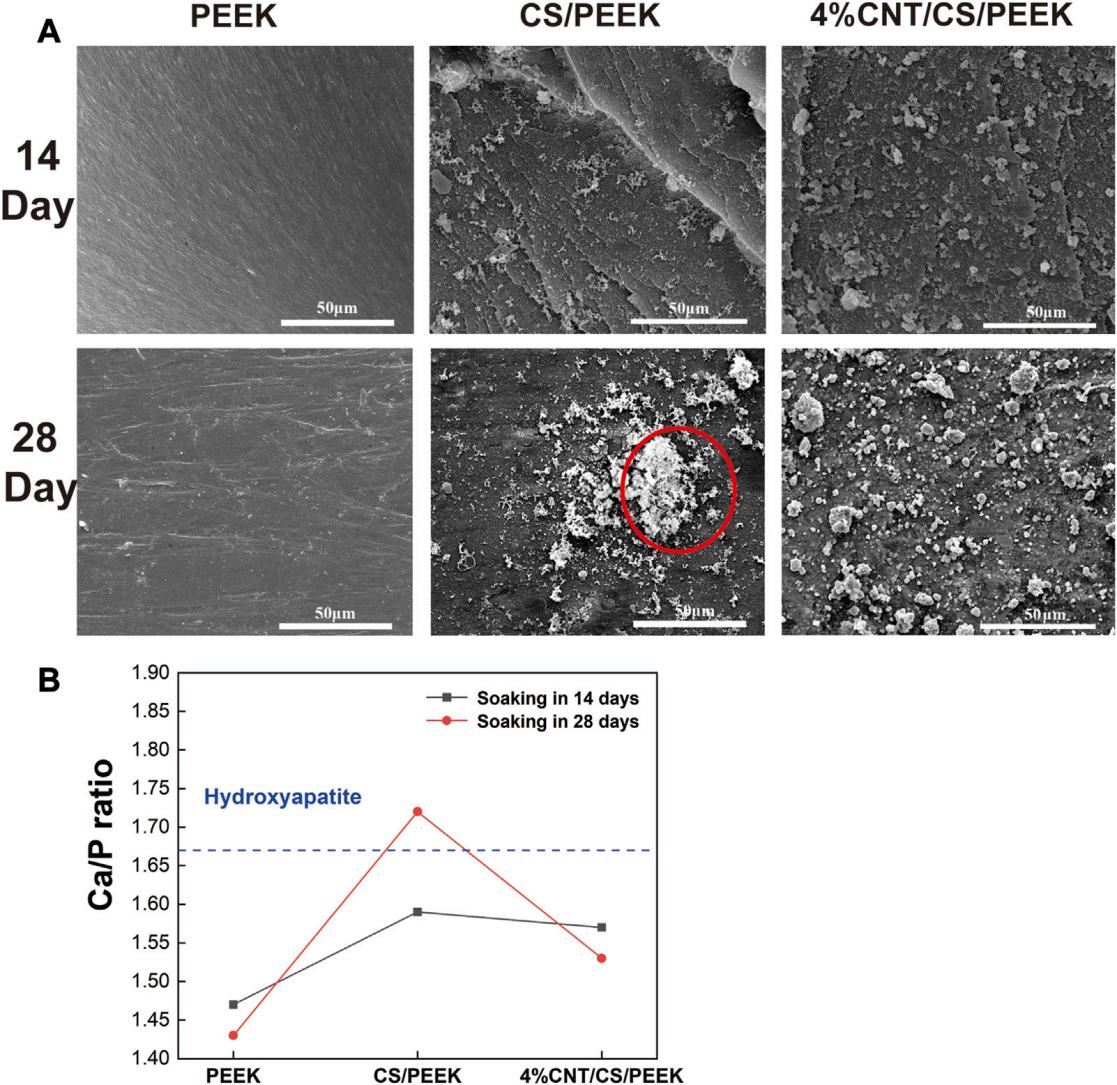
**(A)** Apatite-formation capacity of PEEK, CS/PEEK and 4%CNT/CS/PEEK observed by SEM composites after immersing in simulated body fluid for 14 and 28 days. **(B)** The ratio of Ca/P on the surface of PEEK, and CS/PEEK and 4%CNT/CS/PEEK composites after soaking in SBF for 14 and 28 days.

In general, a formation of a bone-like apatite layer on the surface is the precondition for the effective bonding between biomaterials and living bone *in vivo* (Johnsson and Nancollas, 1992). The biological activity of CS/PEEK and CNT/CS/PEEK composites was evaluated by examining the formation of apatite layer on surface after immersion in SBF (Figure 5A). No obvious difference was observed on the surface of PEEK after soaking for 14 and 28 days, indicating an innate bioinert surface of pure PEEK. In contrast, some flocculent apatites were observed on the surfaces of CS/PEEK and CNT/CS/PEEK composite after being soaked in SBF for14 days. Apparently, after 28 days of soaking, cauliflower-like clusters formed on the surface of the CS/PEEK composites with porous structures which was generated by the dissolution of CS particles (red circles). For ternary CNT/CS/PEEK composites, the entire surface of the composite was also covered with dense globular deposits, forming a tile-shaped and compact coating of apatites. The Ca and P and their ratio of the apatite formed on the immersed specimens was monitored by EDS analysis and the result was presented in Figure 5B. The Ca/P ratio of PEEK was 1.43 after 28 days of immersion in SBF. A higher soaking time in SBF increased the Ca/P ratio of surfaces on CS/PEEK and CNT/CS/PEEK composites. The Ca/P ratios of 1.72 for CS/PEEK and 1.65 for CNT/CS/PEEK samples were obtained respectively after 28 days, revealing nearly identical value to the theoretical Ca/P ratio of hydroxyapatite in 1.67 (Wu and Chang, 2004). Thus, compared to inert PEEK, both the CS/PEEK and CNT/CS/PEEK composites exhibited a superior ability of biological mineralization.

**FIGURE 6:**
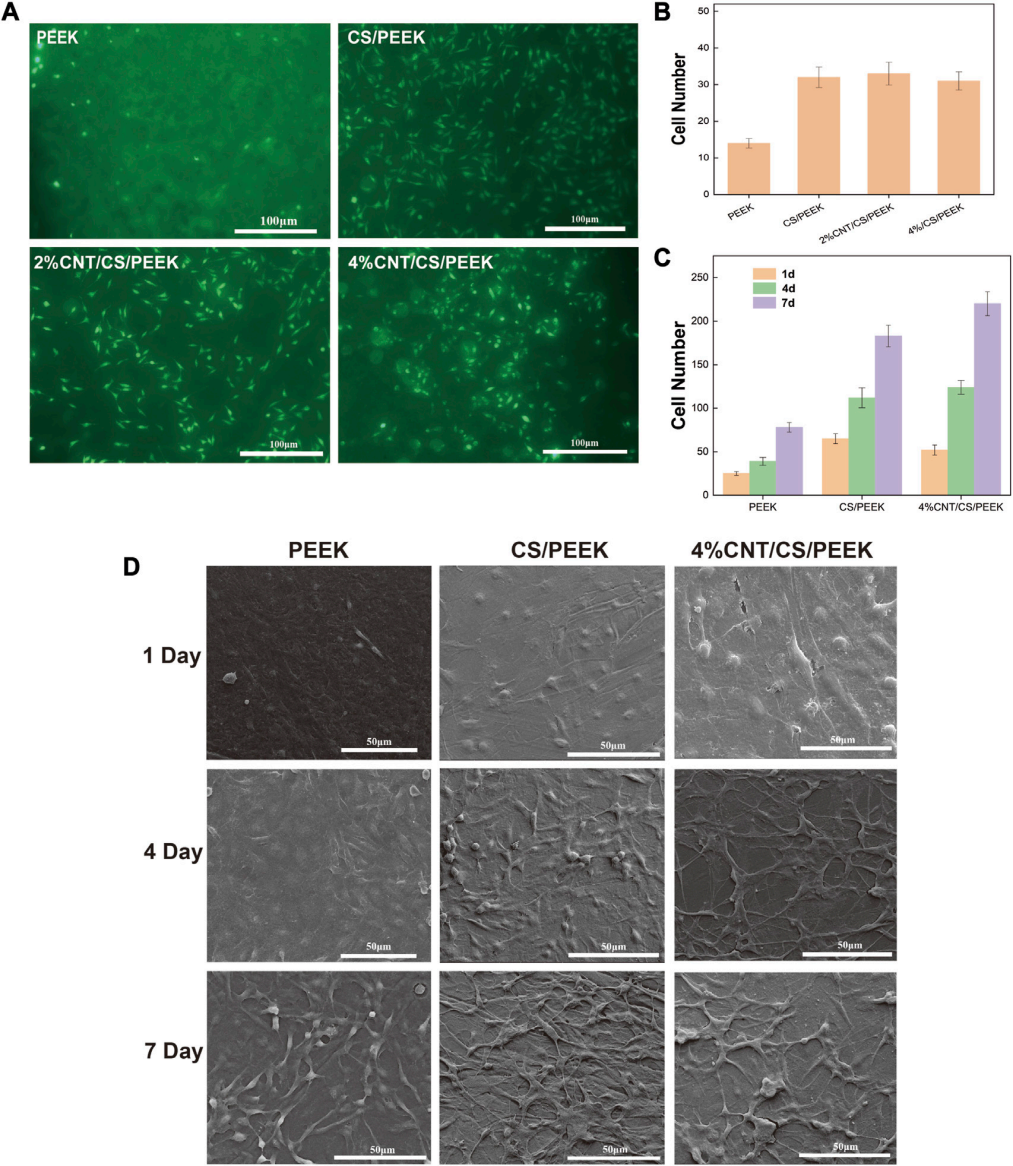
**(A)** Cell adhesion of MC3T3-E1 cells cultured on the surfaces of PEEK, CS/PEEK, 2%CNT/CS/PEEK, 4%CNT/CS/PEEK after incubation for 6 h detected by fluorescence microscopy **(B)** Number of MC3T3-E1 cells on the surface of PEEK and composites after 6 h. **(C)** Number of MC3T3-E1 cells on the surface of PEEK and composites after 1, 4 and 7 days. **(D)** SEM images of the morphologies of MC3T3-E1 cells cultured on PEEK, CS/PEEK composites and 4%CNT/CS/PEEK composites for 1, 4, and 7 days.

Cell adhesion is an necessary requirement for the proliferation and functional differentiation of the osteoblasts (Grzesik, 1997). Figure 6A presented the fluorescence micrographs of MC3T3-E1 cells on the PEEK, CS/PEEK and 4%CNT/CS/PEEK composites after incubation for 6 h. The osteoblasts cells on each specimen exhibited a spindle-shaped or rounded morphologies and suggested the normal cell growth, which proved that PEEK was benign for osteoblasts with a good biocompatibility. Interestingly, more osteoblast cells adhered to the CS/PEEK composite than on PEEK surface, indicating that CS/PEEK composite were more suitable for cell proliferation. The 4%CNT/CS/PEEK composites also showed a higher cell adhesion than PEEK in the early stage. However, as was shown in Figure 6B, no motivation in adhesion was perceived on CNT/CS/PEEK composites when compare to CS/PEEK composite in the first 6 h.

Figure 6D displayed the cell morphologies of MC3T3-E1 osteoblasts on the surface of PEEK, CS/PEEK composites and 4%CNT/CS/PEEK composites after 1, 4, and 7 days of culture. It can be seen that after 1 day of culture, the cells on PEEK surface were sparse but osteoblasts on CS/PEEK and CNT/CS/PEEK composite exhibited a flat type. Besides, cells on CS/PEEK and CNT/CS/PEEK spread faster and extended a number of filopodia protrusions after being cultured for 4 days. Consistently, the osteoblasts proliferated well and formed a confluent layer on the surface of CS/PEEK and CNT/CS/PEEK composites after 7 days. Hence, these results indicated that CS/PEEK and CNT/CS/PEEK composites were conducive to cell attachment and growth of MC3T3-E1 osteoblasts. Furthermore CNT had no negative effects but facilitation on cell proliferation. Figure 6C revealed that cell number and cell proliferation rate adhered on 4%CNT/CS/PEEK increased much faster than PEEK and CS/PEEK composite.

**FIGURE 7:**
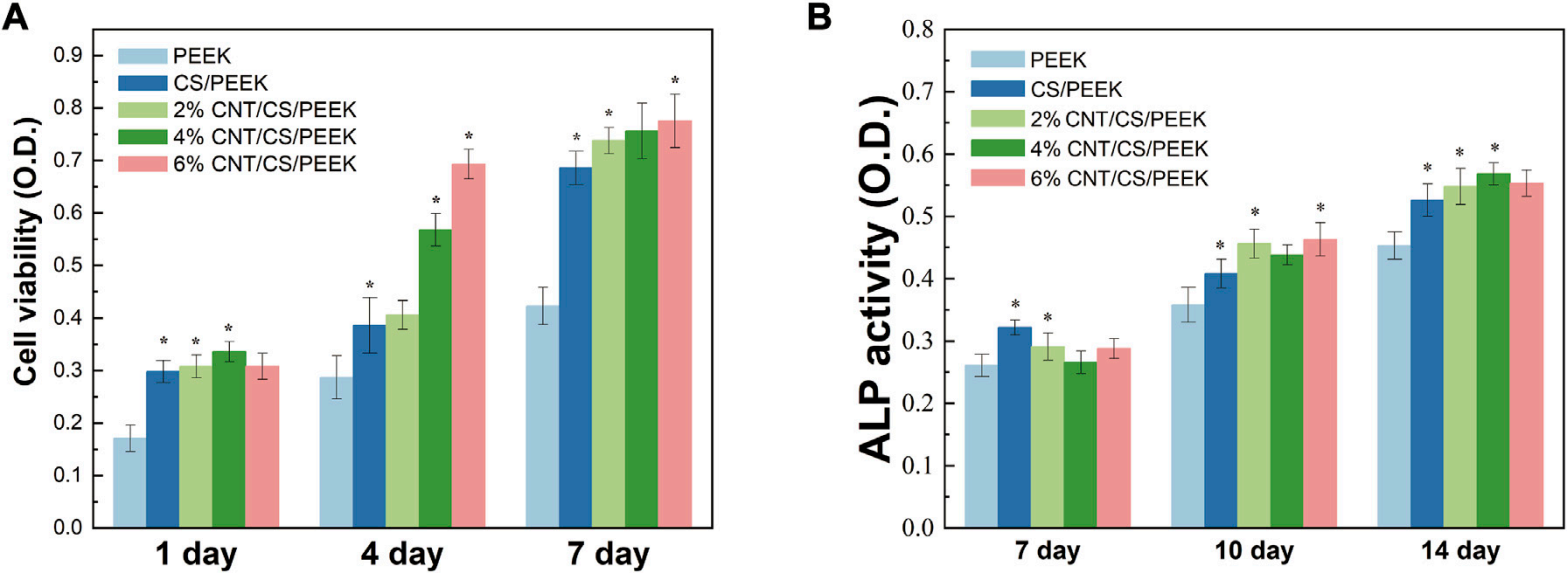
**(A)** Cell metabolic viability of MC3T3-E1cells cultivated on PEEK, CS/PEEK composites and CNT/CS/PEEK composites for 1, 4 and 7 days. **(B)** ALP activity of MC3T3-E1cells cultivated on PEEK, CS/PEEK composites and CNT/CS/PEEK composites for 7, 10 and 14 days. The quantitative analysis ALP activity was normalized to the corresponding content of total protein.

Cell viability was measured by an MTT assay by seeding MC3T3-E1 cells onto PEEK, CS/PEEK composite and CNT/CS/PEEK composites for 1, 4, and 7 days (Figure 7A). In general, cell proliferation on all the specimen proceed as normal because MTT activity on each group were positive-going with increasing incubation time (Zhang et al., 2022). It was interesting to note that PEEK revealed the lowest cell viability among the groups, whereas the OD value of CS/PEEK composite was a significantly higher than PEEK at each detection point, showing that CS promoted the proliferation of MC3T3-E1 cells. In addition, OD value of ternary CNT/CS/PEEK composites was higher than that of binary CS/PEEK composites, and with the increase of CNT content (2 wt%-4 wt%) in composites the activity of cells also boosted after 4 and 7 days. According to the above results, the introduction of CNT and CS improved the cell viability of osteoblasts on PEEK together.

As an extracellular enzyme of osteoblasts, alkaline phosphatase (ALP) and its activity are considered as biomarkers of early cell differentiation due to the increase of ALP activity usual reflects the transition of osteoblasts to a more differentiated state (Liu et al., 2008). Figure 7B showed the ALP activity of the cells on PEEK, CS/PEEK and CNT/CS/PEEK composites at different time. At the end of 7 days, the ALP activity of every group had little difference, which suggested that osteogenic differentiation of the cells was not proceed yet. However, the ALP expression level on the CS/PEEK and CNT/CS/PEEK composites markedly increased at 7 and 10 days. For the CS/PEEK composites, the ALP activity was approximately 13% higher than that of PEEK after 14 days. The ALP activity of CS/PEEK composite was observed to be further increased by adding CNT and the 6%CNTS/CS/PEEK was the highest. At the time point of 10 and 14 days, ALP activity on three CNT/CS/PEEK composites were considerably higher than those on CS/PEEK composite. Based on the experimental results, introducing CS and CNT to PEEK lead to an enhancement in the osteogenic differentiation *in vitro*.

## 4 Conclusion

In this study, ternary CNT/CS/PEEK composites with CS and different CNT contents was successfully fabricated for the first time. The experiment results demonstrated that the addition of 30 wt% CS considerably improved the tensile modulus, bending modulus and microhardness of PEEK. Furthermore, CNT (2 wt% and 4 wt%) was confirmed to compensate for the dramatical mechanical loss caused by friable CS and the mechanical strength of ternary CNT/CS/PEEK composites was even close to that of pure PEEK. The surface hydrophilicity and roughness of PEEK were also improved by adding CS into polymers. Besides, A large-area apatite formed on CS/PEEK and CNT/CS/PEEK composites after soaking in SBF for 28 days which demonstrated the a good biological ability of mineralization. More importantly, on the basis of the results of cell adhesion, proliferation, MTT and ALP assay *in intro*, it was proved that CS and CNT promoted cellular responses and osteogenic differentiation of MC3T3-E1 cells on composites. Therefore, the result suggests that the CNT/CS/PEEK ternary composites might be a promising bone implant material in craniomaxillofacial and orthopaedics surgery thanks to its improved mechanical properties and excellent biocompatibility.

## Data Availability

The original contributions presented in the study are included in the article/Supplementary Material, further inquiries can be directed to the corresponding authors.
